# Short Review on the Biological Activity of Cyclodextrin-Drug Inclusion Complexes Applicable in Veterinary Therapy

**DOI:** 10.3390/molecules28145565

**Published:** 2023-07-21

**Authors:** Mariana Grecu, Bogdan Minea, Liliana-Georgeta Foia, Andra-Cristina Bostanaru-Iliescu, Liviu Miron, Valentin Nastasa, Mihai Mares

**Affiliations:** 1 Laboratory of Antimicrobial Chemotherapy, Faculty of Veterinary Medicine, “Ion Ionescu de la Brad” University of Life Sciences of Iasi (IULS), 8 Mihail Sadoveanu Alley, 700489 Iasi, Romania; mgrecu@uaiasi.ro (M.G.); acbostanaru@uaiasi.ro (A.-C.B.-I.); lmiron@uaiasi.ro (L.M.); mmares@uaiasi.ro (M.M.); 2 Department of Surgery, Faculty of Dental Medicine, “Grigore T. Popa” University of Medicine and Pharmacy of Iasi, 16 Universitatii Street, 700115 Iași, Romania; bogdan-minea@umfiasi.ro (B.M.); georgeta.foia@umfiasi.ro (L.-G.F.)

**Keywords:** cyclodextrin, inclusion complex, antibiotic, antifungal, anti-inflammatory, biological activity, veterinary drugs

## Abstract

Cyclodextrins (CDs) are a family of carrier molecules used to improve the pharmacokinetic parameters of therapeutic molecules. These cyclic oligosaccharides have medical and pharmaceutical applications by being able to form inclusion complexes with molecules that are poorly soluble in water. The benefits of these complexes are directed towards improving the chemical and biological properties—i.e., solubility, bioavailability, stability, non-toxicity and shelf life of drug molecules. Since the 1960s, the first inclusion complexes used in therapeutics were those with α-, β- and γ-CD, which proved their usefulness, but had certain degrees of particularly renal toxicity. Currently, to correct these deficiencies, β-CD derivatives are most frequently used, such as sulfobutylether-β-CD, hydroxypropyl-β-CD, etc. Therefore, it is of interest to bring to the attention of those interested the diversity of current and potential future clinical applications of inclusion complexes in veterinary medicine and to present the contribution of these inclusion complexes in improving drug efficacy. The most important biological activities of β-CD complexed molecules in the veterinary field are summarized in this short review.

## 1. Introduction

Starting from the fact that many of the drugs used in therapeutics have their limitations (unsatisfactory bioavailability and distribution, high toxicity, limited efficacy, active residues, etc.), controlled release systems or carrier molecules have been used to improve their pharmacokinetic profile [1,2]. A group of carrier molecules used in the pharmaceutical industry is represented by cyclodextrins (CDs), obtained by the enzymatic conversion of starch into glucopyranose units organized in a cyclic structure. Structurally, CDs have an inner cavity where various poorly soluble medicinal substances can be included to form soluble inclusion complexes, with great stability both in the solid phase and in the aqueous phase due to the ring structure they possess [3,4].

These characteristics make CDs, and their derivatives have a variety of practical applications in many fields, such as the pharmaceutical industry, medicine, cosmetics, biotechnology, nanotechnology, agriculture, the food industry, the textile industry, the paint industry, etc. However, the most important field of use of inclusion complexes with CDs is the pharmaceutical one in the context of the constant launch of new drugs [5].

In the pharmaceutical industry, CDs are frequently used, mainly due to their ability to significantly increase the solubility of a poorly soluble medicinal substance [6,7], without changing its physical, chemical and biological properties. In medicine, they are used in particular to improve the chemical stability and to increase the bioavailability of drugs [8]. CDs are also used to reduce the irritation caused by the drug at the injection site, to minimize or reduce the unwanted side effects of some drugs at the gastrointestinal or ocular level and for complete renal elimination from the body [9]. At the same time, they are also used to reduce or completely eliminate the unpleasant smell or taste of some active principles, to reduce the incompatibilities and drug interactions that can occur between the active principles and excipients [10,11]. It is also specified that they can be used as potential antidotes in organophosphorus poisoning in animals [12]. CDs prevent drug degradation such as oxidation, hydrolysis, and photodegradation and extend drug shelf life [13,14].

A group of researchers at CycloLab is continuously tracking new drug formulation approvals, particularly those benefiting from the use of CDs. Based on these searches, an internal list containing 129 entries was compiled and occasionally distributed to clients upon request. This list was deemed worthy of further dissemination, so it was turned into an open access scientific paper in the journal *Periodica Polytechnica Chemical Engineering* [15]. Currently, there are more than 50 formulations based on CDs and their derivatives on the market, with their particular structural characteristics, physical and chemical properties, and possible therapeutic applications, but there are very few formulations for the veterinary field in the global context of research on the pharmaceutical use of CDs as carrier molecules [16].

In recent years, these inclusion molecules have been studied on a larger scale in veterinary medicine, but not large enough considering the need to reduce the drug residue that can be found in human food and the adverse reactions commonly encountered in the case of most medicinal substances. As a result of their ability to control in vivo drug release in different biological spaces, the creation of such complexes proves to be an effective tool to improve the efficacy of old and new pharmacodynamically active molecules. However, in veterinary medicine, there is little information available about the real benefits of drugs complexed with CDs, as there are also few such products on the veterinary medicine market.

This short review aims to make known the pharmacokinetic, pharmacodynamic and therapeutic benefits of inclusion complexes for veterinary use.

## 2. Chemistry and Pharmacology Aspects of CDs and Their Inclusion Complexes

One of the major pharmacological properties that characterize the bioavailability of a drug is its solubility in water [17]. The improvement of solubility under physiological conditions is a major requirement for delivery systems designed to raise the efficiency and bioavailability of a drug with poor water solubility [18].

**Figure 1:**
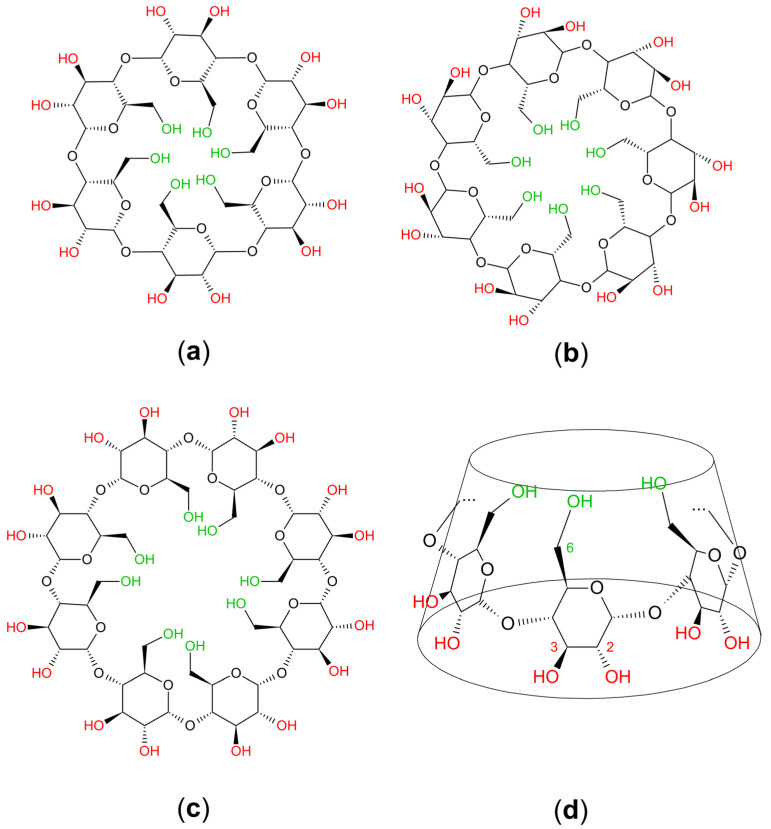
The most common natural cyclodextrins: (**a**) α-cyclodextrin; (**b**) β-cyclodextrin; (**c**) γ-cyclodextrin; (**d**) the toroidal arrangement of the glucose monomers of cyclodextrins.

Cyclodextrins are cyclic oligosaccharides consisting of α-d-glucopyranose units joined by α-1,4 bonds. The most common natural CDs consist of 6 (α-CD), 7 (β-CD) and 8 (γ-CD) units of α-d-glucopyranose (Figure 1a–c). Because free rotation about these bonds is not possible, the CD molecule is not cylindrical, but has the shape of a truncated cone or toroid (Figure 1d) [19].

The X-ray studies revealed that the secondary hydroxyl groups at C2 and C3 (in red) are located on the larger rim of the toroid, while the primary groups at C6 (in green) are on the narrow rim. Apolar hydrogen atoms from C3 and C5 and oxygen atoms from the etheric bonds are situated on the inner side of the ring. The result of this structure is a molecule with a hydrophilic exterior, which can dissolve in water and an apolar cavity that provides a hydrophobic matrix [20,21].

**Figure 2:**
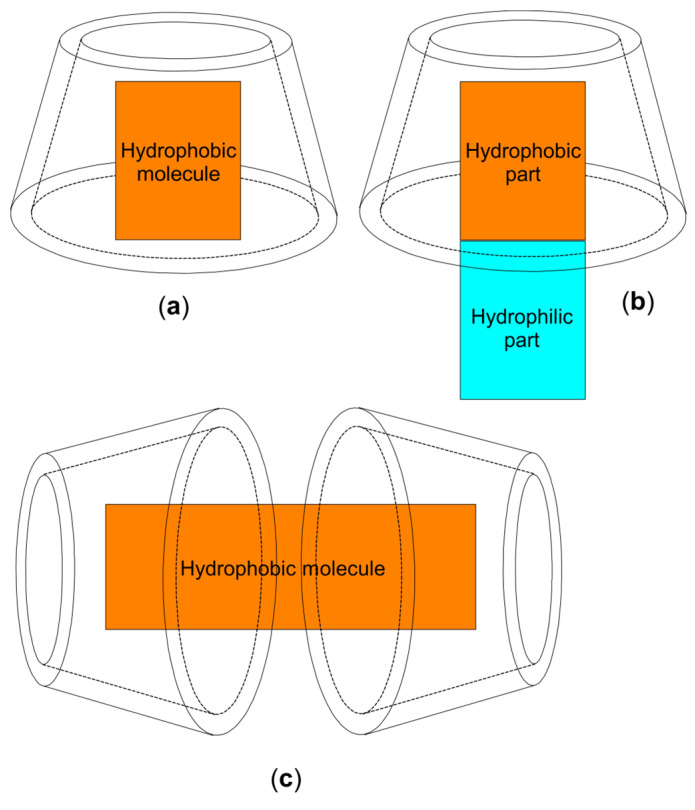
Schematic representation of the most common inclusion modalities: (**a**) complete inclusion of a hydrophobic molecule; (**b**) partial inclusion of only the hydrophobic part of an amphiphilic molecule; (**c**) inclusion into 2 CD molecules of a large hydrophobic molecule.

The cavity of CDs is hydrophobic or, more precisely, only mildly polar and provides a microenvironment where whole hydrophobic molecules (Figure 2a,c) or hydrophobic parts (Figure 2b) of molecules with suitable sizes, can penetrate to achieve complexation [20,22]. In an aqueous solution, the mildly polar CD cavity is occupied by water molecules which can be easily displaced by suitable guest molecules that are less polar. This is the main driving force for complexes formation. The guest forms apolar–apolar associations with the cavity, which results in a more stable state with lower energy [20,23,24]. In addition to the release of water molecules with high entropy, other driving forces for the formation of inclusion complexes are electrostatic, van der Waals and hydrophobic interactions, hydrogen bonds, the decrease of conformational tension (in the case of α-CD), etc. All these forces are relatively weak so that the binding of the guest molecule in the cavity is not permanent. The free drug in the solution is in a rapid equilibrium with the one included in the cavities of CDs [20,21,25].

Due to this relatively apolar cavity, CDs can form inclusion complexes (host-guest complexes) with a large variety of hydrophobic guests both, in solution and in solid state, without forming or breaking covalent bonds [20]. Consequently, in the case of hydrophobic drugs, this encapsulation does not alter their molecular structure or their ability to penetrate hydrophobic biological membranes, but delivers them to the surface of the membranes in their original form [22]. Cyclodextrins improve the bioavailability of insoluble drugs by increasing their water solubility, dissolution and, consequently, their absorption.

The formation of inclusion complexes is a stoichiometric phenomenon in which, usually, only one guest molecule (with a proper size) interacts with the cavity of the CD molecule to become included (Figure 2a,b). In the case of smaller compounds, with low molecular weight, several molecules can fit into the cavity, if its size allows it. In the case of larger compounds, with high molecular weight, several CD molecules can bind to the guest (Figure 2c). In principle, for a complex to form, it is sufficient that only a portion of the guest molecule fit into the cavity. Consequently, molar ratios of 1:1 are not always obtained, especially in the case of guests with high or low molecular weight [20]. Molecules of most drugs form complexes with 1:1 ratio. At lower concentrations of CD, a stoichiometry of 1:1 can occur even with those compounds that normally would have a higher order stoichiometry [19,21].

When a complex is introduced in water, the release of the guest takes place in two stages. The first stage consists in the dissolution of the complex, while in the second stage the guest is released after being displaced by water molecules. An equilibrium occurs between the free and the complexed CD, the guest, the dissolved and the undissolved complex [20]. The improved solubility induced by the complexation increases the efficiency and potency of the active compounds, which reduces the dose required for optimal therapeutic activity. When drugs become effective at lower doses, the therapy risk and side effects are reduced. This increases the pharmaceutical effect and decreases the amount of drug residues. Therefore, CDs can help reduce the toxicity of certain drugs [24,25].

**Figure 3:**
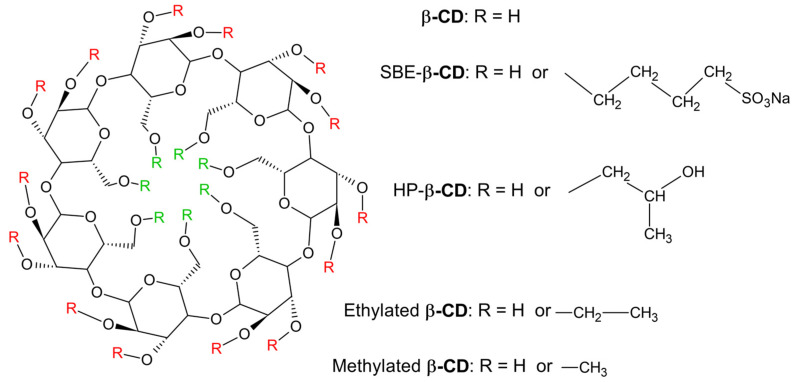
General structure of a β-CD derivative and some of the most commonly used substituents. The substituents (R) attached to the carbons in position 6 of the glucose monomers (located on the narrow rim of the toroid) are green, while those attached to carbons 2 or 3 (located on the large rim of the toroid) are red.

The aqueous solubility of α-, β- and γ-CD is much lower than that of their linear counterparts, most likely due to relatively strong bonds between molecules in the crystalline state. [19,21,23]. The physical and chemical properties and the inclusion ability of the parent CDs, however, can be significantly extended by the addition of a small number of substituents (Figure 3) on some of the hydroxyl groups of the glucose monomers [18,22,24]. These properties, including the complexation ability, can be greatly influenced by the type, number and position of the substituents on the parental molecule. By adjusting these characteristics, CD derivatives with increased water-solubility, stability and with low parenteral toxicity can be obtained [24]. It was found that the substitution of any of the hydroxyl groups, even with hydrophobic groups such as methoxy- or ethoxy-, results in a significant increase in water solubility because the crystalline α-, β- and γ-CDs become amorphous mixtures of isomers as a consequence of the chemical changes. The replacement of more than 2/3 of the hydroxyl groups decreases the number of isomers, which reduces the amorphous character and decreases solubility [19,21,23].

Currently, there are over 1500 synthesized CD derivatives and described in the literature. Due to the high cost of toxicological evaluations, however, only a few of these derivatives are available as pharmaceutical grade excipients [23]. Among all the standardized, industrially produced, and commercially available compounds, the most important in terms of pharmaceutical interest are the hydroxypropyl derivatives of β- and γ-CD (HP-β-CD, HP-γ-CD), sulfobutylether-β-CD (SBE-β-CD) and randomly methylated β-CD [21].

Several factors have to be taken into account when choosing a CD for complexation of a drug. The dimensions of the parental CD dictate what types of guest can be included [20]. Although the cavity of α-CD is insufficient for most drugs, it can form complexes with small molecules or molecules with aliphatic side chains. The cavity of β-CD can include aromatic and heterocyclic rings, while in γ-CD larger molecules, such as macrocycles or steroids, can fit [20,24]. In terms of cost, β-CD is the cheapest of all CDs, while HP-β-CD, SBE-β-CD and γ-CD are more expensive.

Due to their particular properties, the various CD derivatives may offer different drug release profiles. Thus, β-CD derivatives are classified as hydrophilic, hydrophobic or ionizable. Hydrophilic derivatives, such as HP-β-CD or SBE-β-CD, improve the solubility and dissolution rate of poorly water-soluble drugs are useful for immediate release. Hydrophobic compounds, such as ethylated β-CDs, delay the dissolution of water-soluble drugs from their complexes making them potential carriers for a slow and sustained release of drugs with short half-lifes [24,26,27].

In the early stages of pharmaceutical applications, mostly β-CD was used because it was easily available and had a suitable cavity for a wide variety of drugs. Its low water solubility and its nephrotoxicity limited its use, especially for parenteral administration. Cyclodextrin applications in parenteral administration are drug solubilisation, the reduction of irritation at delivery site and the stabilisation of compounds that are normally unstable in water. With a much higher water solubility, HP-β-CD allows the parenteral administration of various drugs without significant toxicity problems and is, therefore, used more often in such formulations. The SBE-β-CD derivative proved to be useful in the preparation of parenteral solutions for positively charged poorly water-soluble drugs. Of all CDs, SBE-β-CD produced the most significant stability increase of many chemically unstable drugs [24].

For oral administration, HP-β-CD proved a better safety profile than β-CD and other parental CDs. All CDs, however, can be considered non-toxic for oral administration, due to the lack of absorption from the gastrointestinal tract. Therefore, due to its low cost, β-CD can be considered optimal for oral administration when its efficiency is sufficient. Modified CD can be used when their higher efficiency and particular properties are required [24].

## 3. The Benefits of Inclusion Complexes (CD–Antibiotic) in Anti-Bacterial Therapy

The discovery of antibiotics was a major milestone in the history of medicine as they saved many human and animal lives. However, the use of these initially miraculous drugs was quickly accompanied by the emergence of antibiotic-resistant bacterial strains, which represent a global public health problem and a serious concern in finding alternative solutions to reduce antibiotic use [28]. Today, the rate at which antibiotic resistance is developing and the rapidity of its spread among different bacterial species is extremely alarming. New forms of resistance to antibiotics spread very easily on all continents, which implies prolonged and more expensive treatments, with sometimes the appearance of severe side reactions and even death.

The treatment of infections in animals can be done either with time-dependent antibiotics (beta-lactams, macrolides, glycopeptides), which require long periods of administration, or with concentration-dependent antibiotics (aminoglycosides, fluoroquinolones), which require high concentrations for antibacterial activity [29]. In both cases, the amounts of residue can be very high and even have a long half-life. Additionally, these antibiotic residues are difficult to remove from food products, they can last a long time (days, months) in the environment and can present a real danger to human and animal health. At the same time, due to the appearance of superbug bacteria, resistant to several classes of antibiotics, these antibiotics lose their therapeutic effectiveness [30].

The development of new antibiotics could not fundamentally solve the problem. The new conventional antibiotics only temporarily improved the situation, because bacteria developed resistance after their long-term use [31]. One of the effective ways that alleviated the resistance of bacteria to antibiotics was their complexation with CDs or their derivatives. Through this technology, an improvement in the therapeutic index (through the controlled release of antibiotics), a reduction in the dosage and the administration interval was achieved [16,32].

Enrofloxacin, a third-generation fluoroquinolone, is a broad-spectrum antimicrobial against many bacterial diseases in animals. The bactericidal activity of enrofloxacin, however, is dependent on the concentration, it has a poor solubility in water, which greatly limits its applicability and therapeutic effect. Wei et al. evaluated through their studies the effectiveness of enrofloxacin and florfenicol complexed with γ-CD. They observed that the new compounds developed much better antimicrobial activity, demonstrated by the fact that the zones of inhibition for E. coli and S. aureus were much clearer [33]. Complexing it with 2-hydroxypropyl-β-CD (HP-β-CD), a CD derivative, resulted in a stable inclusion complex with significantly improved solubility (increased by 32.5%) for enrofloxacin [33,34]. Ding Y. et al. observed a 916-fold improvement in the solubility of complexed enrofloxacin compared to previous studies (169-fold), thus demonstrating that the pharmaceutical, absorption and bioavailability properties of enrofloxacin were significantly modified after complexation with HP-β-CD. The modification of the physical and chemical properties led to the increase of the therapeutic efficacy, to the reduction of the treatment interval, of the amount of antibiotic used and implicitly of the residue, compared to the treatment period of commercial enrofloxacin [34].

The formation of the inclusion complex β-CD–norfloxacin, another fluoroquinolone of the second generation, with antimicrobial effect and broad spectrum of activity—used both in human medicine and in veterinary medicine—was described by Chierentin L. et al., by exploring the structure affinity relationship for norfloxacin in solid and aqueous phases. It was observed that the solubility of norfloxacin was significantly increased by complexation with β-CD. Thermal analysis also showed that the stability of norfloxacin was improved in the presence of β-CD. Microbiological studies have shown that the product complexed with β-CD has a better potency compared to the pure drug due to the improvement of pharmaceutical (stability, dissolution, solubilization), pharmacokinetic (absorption, transport, release and bioavailability) and toxicological (dose and, implicitly, toxic level reduction) properties [35].

Another inclusion complex developed was the one between HP-β-CD and florfenicol, a broad-spectrum antibiotic used in many countries for the treatment of infectious diseases in animals and birds caused by susceptible strains. Since florfenicol has poor solubility in water, it is necessary to use it in large doses, which significantly limits its use. Fan G. et al. analyzing the physical properties of the inclusion complex (Florfenicol–HP-β-CD) compared to simple Florfenicol, observed after intramuscular injection in Beagle dogs, an improvement in pharmacokinetic parameters (elimination half-life [t1/2β], the transport rate constant [K10, K12, K21] and the maximum concentration [Cmax]. In contrast, the half-life of the distribution process [t1/2α], the absorption rate constant (Ka) and the apparent volume of distribution (V1/F) decreased [36]. These results suggest that parenteral administration of Florfenicol–HP-β-CD is promising in antimicrobial therapy, having much higher bioavailability at low doses.

Various other antibiotics used in both human and veterinary medicine have been complexed mostly with β-CD or its derivatives (Table 1), such that by the end of 2021, more than 200 publications discussing the complexation of CDs with antibiotics and other anti-infective agents, including beta-lactams (amoxicillin), tetracyclines (tetracycline, doxycycline), quinolones, macrolides (erythromycin, clarithromycin), aminoglycosides (gentamycin, amikacin), polypeptides (colistin), nitroimidazoles (metronidazole), and oxazolidinones (furazolidone), were registered [16]. These studies focused on improving solubility, altering the drug release profile, slowing drug degradation, improving biological membrane permeability and increasing antimicrobial activity. Although these inclusion complexes are used in human medicine, some of them do have the potential for veterinary use, because the plain non-complexed active drug molecules are already being used in veterinary medicine.

## 4. Benefits of Inclusion Complexes (CD–Antifungal) in Antifungal Therapy

Fungal infections are relatively rare in animals, being found especially in dogs and cats in the form of dermatological skin diseases, called dermatophytoses. Dermatophytes are the group of fungi that feed on keratin and rarely penetrate under the skin. Species adapted to animal hosts can occasionally be transmitted to humans with whom they come into contact or to other animal species. The main dermatophytes of interest for veterinary medicine are from the genera *Epidermophyton*, *Microsporum* and *Trichophyton* [38].

Fungal infections are often extremely difficult to treat, given the limited number of drugs for animals and even for humans. Depending on the strategy chosen, topical and/or systemic drugs can be used based on the clinical picture of the host and identification of the etiological agent. For effective treatment, it is important to correctly identify the causative agents at the species level, which will allow the initiation of appropriate therapy. Usually, the management of fungal infections in animals involves combined systemic and topical treatments, and decontamination of the environment in which the animal lives. The resolution of fungal infections requires the institution of systemic therapy, because fungi, like bacteria, develop mechanisms by which they combat the fungicidal or fungistatic effects of antifungal agents and induce resistance.

Only a few products are licensed for animals, and as a result, off-label use of drugs approved for human use is quite common [39]. The commercial availability of antifungal agents is limited mainly by economic, pharmacokinetic (low bioavailability), therapeutic (long-term treatments of weeks, months and even years), and toxicological considerations (hepatic adverse effects and a small therapeutic index), aspects that have imposed finding new solutions for classic antifungals with the aim of significantly reducing their toxicity and improving their therapeutic efficacy. Thus, the complexation with CDs proved to be one of the most successful options [40].

Certain studies pointed out that the acute toxicity of flucytosine–β-CD and sulconazole–β-CD complexes was much lower compared to classical drugs [41,42]. There was also an improvement in the dissolution rate after complexation, making the new compounds viable candidates for antifungal therapy. These results recommend the described conjugates as promising therapeutic agents in the future for all animal species with fungal diseases.

Farooq M. et al. demonstrated that by complexing voriconazole with HP-β-CD, no acute toxicity phenomena were observed after oral administration in experimental animals [43]. Another study investigated the clinical effects of voriconazole complexed with SBE-β-CD administered intravenously in 25 human patients with invasive aspergillosis. The obtained results highlight the safety of treatment with complexed voriconazole from the point of view of toxicity, none of the patients presented a significant deterioration of renal function, an aspect recorded even in patients with renal failure [44]. We can conclude that the development of inclusion complexes can be a promising tool to improve the solubility and efficacy of hydrophobic drugs, such as voriconazole, in order to reduce toxicity and effectively manage fungal infections.

Another triazole antifungal complexed with CDs and widely used in the therapy of fungal infections in companion animals was itraconazole [45,46,47,48]. Classic itraconazole treatment in carnivores has often been associated with liver, heart, kidney and digestive disorders. [49,50]. The USA Food and Drug Administration (FDA) approved an inclusion complex consisting of itraconazole and HP-β-CD for the therapy of dermatophytosis in companion animals. This is a generic product that, in both cats and dogs, shows much improved absorption and bioavailability compared to plain itraconazole [51,52]. It was also found that this inclusion complex has a greater antifungal action than fluconazole and ketoconazole, it is much safer, cheaper and more economical to use than the new triazoles [53,54,55]. It seems that the positive results recorded during the research made this complex today the antifungal of choice in the therapy of fungal infections in pets.

An experimental complex of β-CD with propiconazole nitrate (PCZ-NO_3_), a propiconazole-derived triazole with antifungal effect, was investigated. The complex, generically named MXP-4509, was stable, water-soluble, thus allowing its injectable administration. To assess the antifungal activity of MXP-4509, tests were performed both in vitro on Candida yeasts in planktonic and biofilm form, and in vivo on murine models of disseminated candidosis. The inclusion complex showed excellent antifungal activity in vitro. The distribution of the MICs for the complex showed a significant difference for Candida spp., being lower vis a vis the MICs of fluconazole and similar to those of voriconazole. Therapeutically, the inclusion complex MX-4509 may have a pharmacokinetic and pharmacodynamic profile similar to voriconazole [56]. 

As azoles in general have a relatively poor water solubility, most of them would potentially benefit from complexation with a β-CD, including some that are already used in veterinary therapy in their plane non-complexed form (Table 2).

## 5. Benefits of Inclusion Complexes (CD–NSAIDs; CD–SAIDs) in Pain Therapy

Osteo-articular inflammatory conditions are known to induce physical and even psychological discomfort in both humans and animals due to intense pain and limitation of movement and motion. Non-steroidal anti-inflammatory drugs (NSAIDs) are one of the most widely used classes of drugs in the treatment of inflammation, fever and pain. Therapy of these conditions with NSAIDs requires a long period of administration, but induces significant digestive, renal or cardiac toxicity [59,60]. 

Because of this situation, in recent years several studies focused on finding new molecular targets and new compounds to reduce adverse reactions and for better efficacy of pharmacodynamically active molecules. The inclusion of old molecules into CDs and their derivatives (Table 3), however, has also led to promising results, with the CD host molecule providing protection to the incorporated NSAID and significantly reducing some of the adverse effects that the active substance typically causes [9].

A systematic search of papers published between 2010 and 2020 was performed by Miranda et al. using the Preferred Reporting Items for Systematic Reviews and Meta-Analyses (PRISMA) protocol as well as various search terms (complexation”, “cyclodextrin”, “nonsteroidal anti-inflammatory drug”) [61]. They identified a number of 24 different NSAIDs that were complexed with CDs, such as meloxicam, diclofenac, flurbiprofen, ibuprofen, piroxicam, aceclofenac and oxaprozin. They observed that 12 types of CD were used, and 60 distinct inclusion complexes were formed, of which meloxicam complexed with β-CD appeared in most studies. These results show that CDs are drug delivery systems capable of enhancing the pharmacological and biopharmaceutical properties of nonsteroidal anti-inflammatory drugs.

Ketoprofen [62,63], which is commonly used in colic in horses, tolfenamic acid [64], meloxicam [65,66], carprofen [67] or piroxicam [68], which are used in dogs and cats to relieve pain and inflammation associated with osteoarthritis, complexed with β-CD or HP-β-CD ultimately resulted in increased bioavailability with improved analgesic and anti-inflammatory activity and decreased ulcerogenic effect.

Alsarra et al. proved in their studies that β-CD and HP-β-CD forming compounds with different non-steroidal anti-inflammatory drugs were safe for oral use [69]. Cyclodextrins act as protective agents against gastrointestinal disorders, each of these CDs can be used in the formulation of oral preparations of indomethacin or piroxicam to avoid the typical side effects of ulceration.

Tolfenamic acid is an anti-inflammatory commonly used in veterinary medicine for dogs and cats to combat osteo-articular and musculoskeletal inflammation and pain, as well as to combat febrile syndromes. Stasiłowicz et al. described in their studies that tolfenamic acid, a practically insoluble substance, dissolved in 180 min upward of 80% by combining with β-CD, and upward of 90% with HP-β-CD, which resulted in better analgesia compared to the same dose of plain tolfenamic acid [64].

Anti-inflammatory drug complexes with CDs seem to hold a prominent position today among the significant number of CD-based formulations that have been studied or are on the market. The important achievements of anti-inflammatories obtained by complexation with CDs triggered intense research over the years [70,71,72] and led to their more intensive use in the therapy of inflammatory conditions or as analgesics in animals.

Many other studies have used CDs (especially β-CD and HP-β-CD) to improve the water solubility and increase the bioavailability of NSAIDs [69], It can be mentioned that the increased solubility was associated with an improved bioavailability [73], with an effective analgesia, with increased anti-inflammatory activity, but also with the reduction of adverse reactions [62]. Therefore, strategies aimed at improving the safety and efficacy of NSAIDs, such as CD preparations, have a fundamental impact on anti-inflammatory therapy.

Cyclodextrins have also been complexed with glucocorticoid steroidal anti-inflammatory drugs (Table 3), which represent one of the most prescribed classes of drugs worldwide, with an indisputable effectiveness in the treatment of acute or chronic inflammation, allergic diseases, acute pathological conditions (anaphylactic shock, pulmonary edema, COVID-19, etc.). Glucocorticoids have been used for over 70 years as a safe and effective treatment option in rheumatoid arthritis in dogs, autoimmune diseases in dogs and cats, respiratory allergies in horses, atopic allergies in dogs and cats. However, their long-term use has led to the development of adverse effects, such as digestive damage, high blood pressure, and metabolic changes [74]. The most used drugs in this class are dexamethasone and prednisolone, both of which are synthetic and have a low solubility in water, which makes their formulation difficult [75].

In vitro research, preclinical and clinical studies on the use of glucocorticoids in animals were considered by Santos et al. for a systematic review of their effect vis-à-vis the efficacy of CD–glucocorticoid complexes, compared to the uncomplexed drug, in terms of inhibiting inflammatory mediators and reducing edema [73]. For example, Tan, et al., studying in vitro a complex of hydrocortisone acetate–βCD, specified that the solubility, stability and release of the drug were much higher than that of plain hydrocortisone acetate [76]. Dexamethasone coupled to HP-β-CD induced a marked reduction in inflammation with improved vision in uveitis-affected rabbits [77]. Prednisolone, another SAID with significant veterinary use, showed increased dissolution rates and enhanced bioavailability after complexation with β-CD [78]. 

Therefore, the conjugation of NSAIDs and SAIDs with CDs leads to major changes by improving the therapeutic properties and, above all, to reducing the adverse effects usually encountered in the treatment with these classic molecules. Coupling with these substances has indisputable advantages in terms of changing the pharmacokinetic profile of these molecules, among the most used in veterinary medicine. For example, in a survey of 2000 veterinarians who had in their custody farm animals intended for human consumption, it emerged that 57% of those who responded (91%) use this group of drugs more than 2 times per week in their current practice [79]. This study shows the extent of use in animals (not including pets) of this group of drugs, as well as the usefulness and the need to look for solutions to effectively improve their pharmacokinetic and pharmacodynamic profile.

**Table 3:** Brief overview of the inclusion complexes of NSAIDs/SAIDs used in veterinary medicine.

Active Compound	Pharmacological Activity	Treated Species	CD Types	Characterization	Stoichiometry Guest: Host	References
Ketoprofen	Analgesic, anti-inflammatory and antipyretic properties; acute or chronic inflammation	horse, cattle, sheep and goats, pigs	β-CD	XRD, Dissolution Studies	1:1	[62,63]
Tolfenamic acid	Acute and chronic inflammatory conditions, fever, postoperative pain	cattle, pig, dog and cat	HP-β-CD	XRD, FT-IR	1:1	[64]
Meloxicam	Acute and chronic musculoskeletal pain, post-operative inflammation; acute respiratory infection and postoperative pain	dog, cat and cattle	β-CD	HPLC, Dissolution Studies	1:1	[65]
Carprofen	Acute and chronic musculoskeletal disorders (osteoarthritis); post-operative analgesic; acute respiratory infections	dog, cat and cattle	HP-β-CD	TEM, HPLC, DSC	1:1	[67]
Piroxicam	us ‘off label’ or ‘extra label’ in bladder transitional cell carcinoma, as well as other cancers	dog and cat	β-CD	DSC; XRD; HPLC	1:2.5	[68]
Dexamethasone	Anti-inflammatory effect in inflammatory and immune-mediated disease; diagnostic test of adrenal function	all animal species	HP-β-CD	UV-spectroscopy, Dissolution Studies	1:1	[77,80]
Hydrocortisone-acetate	Adrenal-gland dysfunction (Addison’s disease); Inflammatory conditions	dogs and cat	β-CD HP-β-CD	XRD, DSC	1:1	[76,81]
Methyl-prednisolone	Supportive treatment in ketosis, rheumatoid arthritis, bursitis inflammatory and allergic conditions	large and small animals	β-CD γ-CD	DTA; XRD, H-NMR	1:2 2:3	[78,82]

## 6. Other Inclusion Complexes Used in Veterinary Therapy

In recent years, research related to the phenomenon of inclusion in veterinary medicine has gained momentum, with several commercially known formulations with pharmacodynamically active molecules specific to this field.

Alfaxalone (Alfaxan™) is a synthetic neuroactive steroid used in veterinary practice to induce and maintain general anesthesia in dogs and cats [83,84]. The anesthetic properties of alfaxalone have been known since 1971, but due to adverse reactions it was withdrawn from the market, after which it returned in 2001 in a new formulation. In the new presentation, it was complexed with HP-β-CD aiming to increase solubility and bioavailability [85,86,87,88]. 

Maropitant (Cerenia^TM^) is an NK_1_ antagonist antiemetic approved by the FDA for the treatment of motion sickness and vomiting in dogs and cats. One of the most common side effects of subcutaneous administration is moderate to severe pain at the injection site [89]. By coupling it with sulfobutylether-beta-CD this adverse effect was resolved by increasing its solubility and conditioning it at low temperatures, recording a significant attenuation of pain at the injection site [90,91,92]. Intravenous pain has not yet been evaluated, but rapid administration of a dose induces a temporary drop in blood pressure [91] and rarely anaphylactic reactions in dogs and cats [93]. Probably, the discovery of new carrier molecules would lead to solving many of these side effects in the futures.

Another modified molecule is Pimobendan (Vetmedin™), a drug used in the treatment of heart failure in dogs, with positive inotropic and vasodilator effects. The water solubility of pimobendan depends on pH, being significantly higher at pH 1–3, however, the chemical stability in solution is reduced, so it is impossible to obtain a stable solution with a reasonable shelf life. In addition, the local tolerance of such a formulation is very poor. By complexing pimobendan with ethylated derivatives of β-CD, its solubility and safety in parenteral administration were greatly improved [94].

## 7. Conclusions

On closer inspection, many of the studies published today are aimed at developing new therapeutic systems based on existing active pharmaceutical ingredients with known safety profile and physical and chemical characteristics. Because of the high costs, long time to market, and possibly huge risk of failure in clinical trials, in the development of new molecules in the veterinary field today, a good opportunity turns out to be the development of new administration systems that use molecules that can modify the unfavorable physical and chemical properties of other already known pharmacodynamically active molecules. In this sense, CDs have undoubtedly been very successful, a fact revealed by the formulations described and currently existing on the veterinary market.

These new formulations have conferred new properties to poorly soluble medicinal substances, by increasing solubility, bioavailability, distribution, elimination, reduction of adverse effects, dosage, the time required to carry out the treatment and ease of handling the medicine.

## Figures and Tables

**Table 1:** Brief overview of the inclusion complexes of antibacterial drugs used in veterinary medicine.

Active Compound	Activity Spectrum	Treated Species	CD Type	Characterization	Stoichiometry Guest: Host	References
Enrofloxacin	*Staphylococcus*, *Escherichia coli*, *Proteus*, *Klebsiella*, *Pasteurella multocida*, *Pseudomonas*, *Rickettsia*, *Chlamydophila felis*, *Actinobacillus pleuropneumoniae*, *Haemophilus parasuis*, and *Streptococcus suis*, *Mannheimia haemolytica* and *Haemophilus somni*	all animal species	γ-CD	FT-IR, 1H-NMR, SEM, UV spectroscopy, HPLC, Dissolution Studies	1:1	[34,37]
Norfloxacin	*Mycoplasma*, Gram-positive (staphylococci, streptococci, etc.) and Gram-negative (colibacilli, *Pasteurella* spp., *Salmonella* spp.)	cattle, sheep, goats, pigs and birds	β-CD	DSC, TGA, FT-IR, XRD, SEM, NMR spectrometry, HPLC, Dissolution Studies	1:1	[35,37]
Florfenicol	Gram-positive bacilli and Gram-negative cocci and *Mycoplasma*	cattle, sheep and pigs	HP-β-CD	SEM, XRD, DSC, FT-IR, 1H-NMR	1:1	[36,37]
Amoxicillin	Gram-positive bacteria, in particular streptococcal bacteria causing upper respiratory tract infections	all animal species	HP-β-CD	MDS, IMC, MM, HPLC	1:1	[16,37]
Gentamicin sulphate	Aerobic Gram-negative bacteria (e.g., *Escherichia coli*, *Klebsiella pneumoniae*, *Serratia* spp. and *Enterobacter* spp.), *Pseudomonas aeruginosa*, and some strains of *Neisseria*, *Moraxella*, and *Haemophilus*	horse, foal, cattle, calf, pig, dog, cat	β-CD	SEM, FT-IR, TGA	n/a	[16,37]
Metronidazole	Protozoans (*Entamoeba histolytica*, *Giardia lamblia* and *Trichomonas vaginalis*) and most Gram-negative (*Bacteroides* and *Fusobacterium*) and Gram-positive (pepto-streptococci and *Clostridia* spp.) anaerobic bacteria	dogs and cats	HP-β-CD	Rheology, SEM, NMR, FT-IR, DSC, TGA, XRD, Dissolution Studies	n/a	[16,37]

**Table 2:** Brief overview of the inclusion complexes of antifungal drugs used in veterinary medicine.

Active Compound	Activity Spectrum	Treated Species	CD Type	Characterization	Stoichiometry Guest: Host	References
Flucytosine	*Cryptococcus neoformans*, *Candida* spp. and filamentous fungi like *Aspergillus* spp.	dog and cat	β-CD HP-β-CD	UV–VIS, 1H-NMR, Dissolution Studies, DSC, SEM, FT-IR, XRD	1:1	[37,42]
Sulconazole nitrate	*Trichophyton rubrum*, *Trichophyton mentagrophytes*, *Epidermophyton floccosum*, and *Microsporum canis* and *Malassezia furfur*	dog	β-CD	1H-NMR, DSC, TGA, SEM, XRD	1:1	[37,41]
Voriconazole	*Blastomyces* spp., *Cryptococcus neoformans* and *C. gattii* and aspergillosis (*A. fumigatus*, *A. terreus*, *A. flavus*, f *A. nidulans*)	dog, rarely in cat, horse, cow ferret, deer, bird, many wildlife species	HP-β-CD SBE-β-CD γ-CD	SEM, FT-IR, DSC, XRD, Dissolution Studies	Variable, from 1:2 to 1:4	[37,43,44,57,58]
Itraconazole	*Cryptococcus neoformans* and *C. gattii*, *Microsporum canis*, *Trichophyton* spp., *T. terrestre* and *Microsporum gypseum*.	dog, horse, bird, small mammals, and reptiles.	β-CD HP-β-CD	FT-IR, DSC, UV spectroscopy, Dissolution Studies	1:2	[37,47,48]

## Data Availability

No new data were created or analyzed in this study. Data sharing is not applicable to this article.

## Abbreviations

**1H-NMR**: 1H Nuclear Magnetic Resonance;	**NSAID**: Non-Steroidal Anti-Inflammatory Drug
**C**: carbon atom	**SAID**: Steroidal Anti-Inflammatory Drug
**CD**: cyclodextrin	**SBE-β-CD**: sulfobutylether-β-cyclodextrin;
**DSC**: Differential Scanning Calorimetry;	**SEM**: Scanning Electron Microscopy;
**DTA**: Differential Thermal Analysis	**TEM**: Transmission Electron Microscopy
**FT-IR**: Fourier-Transform Infrared Spectroscopy;	**TGA**: Thermogravimetric Analysis;
**HPLC**: High Performance Liquid Chromatography	**UV–VIS**: Ultraviolet-Visible Spectroscopy;
**HP-β-CD**: hydroxypropyl-β-cyclodextrin;	**XRD**: X-ray Diffraction Analysis;
**IMC**: Isothermal Microcalorimetry;	**α-CD**: alpha-cyclodextrin;
**MDS**: Middle Distillate Synthesis;	**β-CD**: beta-cyclodextrin;
**MM**: Molecular Modelling;	**γ-CD**: gamma-cyclodextrin;
**n/a**: not available;	
